# The Recent-Transmission of Mycobacterium tuberculosis Strains among Iranian and Afghan Relapse Cases: a DNA-fingerprinting using RFLP and spoligotyping

**DOI:** 10.1186/1471-2334-8-109

**Published:** 2008-08-06

**Authors:** Parissa-Farnia, Mohammad Reza Masjedi, Mohammad Varahram, Mehdi Mirsaeidi, Mojtaba Ahmadi, Mehdi Khazampour, Payam Tabarsi, Parvaneh Baghei, Mojtaba Marjane, Muslam Bahadori, Abolhasan Zia Zarifi, Ali Akbar Velayati

**Affiliations:** 1 Mycobacteriology Research Centre, Iranian National Reference TB Laboratory, National Research Institute Of Tuberculosis and Lung Disease (NRITLD), Shaheed Bahesti University of Medical Sciences(Medical Campus), Shaheed Bahonar Ave, Darabad, Tehran, 19556, P.O: 19575/154, Iran

## Abstract

**Background:**

Relapse of tuberculosis (TB) may develop as the result of reactivation of the endogenous primary infection, or as a result of a exogenous reinfection. This survey evaluated the rate of reactivation versus recent transmission among Iranian and Afghan relapse cases.

**Methods:**

The sputum specimens were digested, examined microscopically for acid-fast bacilli, and inoculated into Löwenstein-Jensen slants by standard procedures. Thereafter, the susceptibility and identification tests were performed on culture positive specimens. Subsequently, the strains that were identified as *Mycobacterium tuberculosis *(258 isolates) were subjected to IS6110 restriction fragment length polymorphism (RFLP) and spoligotyping. Additional patient's information was collected for further epidemiological analysis. Patients whose isolates had identical genotyping patterns were considered a cluster with recent transmission episode.

**Results:**

Out of 258 available isolates, 72(28%) had multi-drug resistant (MDR-TB) in ratio and 42 (16.2%) had other resistant. Notably, 38 of MDR-TB cases (52%) were isolated from Afghan patients. By IS6110-RFLP typing method, 65 patients (25%) were clustered in 29 clusters. In cluster cases, the intra-community transmissions between Iranian and Afghan patients were 41%. All MDR-TB patients in clusters had either Haarlem I or Beijing characteristic. The risk factors like sex, family history, close contact, living condition, PPD test result and site of TB infection were not associated with clustering. Although, the MDR-TB strains were more frequent in non-cluster cases (31%) than cluster one(18%) (P < 0.05). Majority of *M. tuberculosis *strains isolated from non-cluster cases were belong to EAI3 (51; 30%) and CASI(32;18.6%) superfamilies.

**Conclusion:**

During the studied period, reactivation of a previous infection remain the more probable cause of recurrence. Although, the evidence of intra- community transmission between Iranian and Afghan TB cases, highlighted the impact of afghan immigrants in national tuberculosis control program (NTP) of Iran.

## Background

Restriction fragment length polymorphism (RFLP) using insertion sequence IS6110 is a well-established method of "DNA fingerprinting" that has been used to trace the transmission of particular strains of *M. tuberculosis *isolates [1,2]. The method is based on the detection of differences in the numbers and locations of the insertion element IS6110 within the chromosomes of *M. tuberculosis *strains [2,3]. Generally, the unrelated clinical isolates show a high degree of variation, whereas, epidemiologically related strains show identical or similar fingerprint patterns [4,5]. By IS*6110*-RFLP, it was also possible to determine whether a new episode of disease is caused by reactivation of endogenous infection or by exogenous reinfection [6,7]. The exogenous reinfection can either occur during therapy for the original infection or after therapy has been completed [8]. In overall, the frequency with which the patient reinfected with *M. tuberculosis *are reported to be variable. In South Africa, the frequency of exogenous reinfection reported to be high, whereas in other places it has been involved selected populations e.g., alcoholic residents of a homeless shelter or patients with advanced HIV infection[8,9]. In Iran, the extent in which exogenous reinfection contributed to incidence of the diseases are not known. According to the World Health Organization, the estimated incidence of TB in Iran is 28 cases per 100000 populations [10]. The TB problem has become more serve because of an increase in MDR-TB strains. Based on national wide survey conducted in 1999, among all *M. tuberculosis *isolates tested for drug susceptibility, 10.9% were resistant to ≥ 1 anti-TB drug, and 6.7% were resistant to both isoniazid and rifampin (i.e., were MDR strains of *M. tuberculosis*)[11]. In further studies, the existence and transmission of XDR-TB strains (i.e., resistant to fluoroquinolones and to at least one of the three inject able second line drugs in addition to isoniazid and rifampin) in epidemiological related MDR-TB patients were demonstrated [12]. Considering the severity of diseases associated with spread and transmission of MDR or XDR-TB strains, we tried to determine the relative frequency of reactivation from recent transmission among relapse cases using IS6110-RFLP. However, since RFLP analysis with IS6110 alone may be inconclusive for strains carrying few copies of IS6110 [13], we also used an alternative PCR-technique called spoligotyping. The technique detects various non-repetitive spacer sequences located between small repetitive units (direct repeat DR) in the chromosome on *M. tuberculosis *complex [14]. In present study, the contribution of Afghan immigrants in maintaining the recurrent tuberculosis was also determined.

## Methods

### Setting

The National Research Institute of tuberculosis and Lung Diseases (Tehran/Iran), which acts as the reference unit for National Tuberculosis Program, is the only centre for diagnosis and treatment of MDR and relapse TB patients.

### Patients

Patients included in this study had at least two episodes of TB, with cure as the outcome of the first episode. According to WHO criteria, cure was defined as the completion of a course of six to eight months of directly observed combination therapy (with isoniazid, rifampin, and pyrazinamide in a single tablet), compliance (attendance for the course of therapy, with at least 80 percent of prescribed doses taken), and a sputum culture positive for *M. tuberculosis *at diagnosis and at least one negative sputum culture at the end of treatment. Recurrence or relapse was defined as development of a culture positive for *M. tuberculosis *and symptoms consistent with tuberculosis after the patient had completed a course of treatment and had been confirmed culture negative and clinically recovered [11].

### Data collection

The study was conducted from June 2006 to June 2007. Generally, all heath facilities in Tehran refer their TB suspect to National Reference TB laboratory (NRL) Tehran, Iran for susceptibility and identification test. Case data were collected by trained technicians using standard questionnaires. Information was obtained on sex (female & male), age, contact (family contact/close contact), previous TB history, present address and associated medical data such as HIV infection (yes, no, not known), and tuberculin skin test (+, -, equivocal). The patients with similar or highly similar IS6110-RFLP fingerprint patterns were interviewed together. The Institutional Review Board at the National Research Institute of Tuberculosis and Lung Diseases in Tehran approved the study.

### Bacterial strains

Primary isolation and culturing of *Mycobacterium *isolates from sputum specimen were followed in accordance to procedures manual [15]. All isolates were identified as *M. tuberculosis *by using biochemical tests, including production of niacin, catalase activity, nitrate reduction, pigment production and growth rate [15]. Drug susceptibility testing against isoniazid (INH), rifampicin (RF), streptomycin (SM), ethambutol (ETB) and pyrazinamide (PZA) were performed by the proportional method on Löwenstein-Jensen media at a concentration of 0.2,40,4.0 and 2.0 μg/ml, respectively [16].

### IS6110-RFLP typing

DNA extraction, digestion and southern blotting were performed by standard protocols[1,2]. 5–10 ng of chromosomal DNA was digested with 2 unit/μl of PVUII restriction enzyme and was hybridized with a 10 μl of probe which prepared from a 245 bp PCR product of IS6110.

### Spoligotyping

The method was performed as previously described by Kamerbeek et al [17]. In brief, DR region was amplified by PCR using primers derived from the DR sequence. The amplified DNA was hybridized to a set of 43 immobilized oligonucleotides derived from the spacer sequences of M. tuberculosis H37RV and M. Bovis BCG P3 by reverse line blotting.

### Computer-assisted analysis of fingerprints

The autoradiograph of IS6110-RFLP and spoligotyping were scanned with Snap Scan 1236 Scanner. Bionumerics Software (version 2.5, Applied Math's) was used to analyze the molecular patterns generated by IS6110-RFLP and spoligotyping. The dendograms were generated by the hierarchic unweighted pair group method analysis (UPGMA) clustering algorithm. (The UPGMA used because the distance between two clusters was calculated as the average distance between all pairs of objects in two different clusters). Strains were classified in a cluster when they shared similar IS6110-RFLP and spoligotyping patterns.

### Statistical analysis

The continuous variables were expressed as group means ± SD. The Variables were included sex, age, Family/close contact, pattern of drug resistance, PPD test, between the groups of tuberculosis patients in cluster (consider as a recent transmission) and non-cluster cases (consider as a reactivation).

## Results

### Particulars of the Patients

The study involved 199; 77.1% Iranian and 59; 22.8% afghan cases. The median age was 47 and 38 years for Iranian and Afghan TB patients, respectively (table. 1). 111(43%) were female and 147(56.9%) were male. The male to female ratio was 1.3:1. As shown in table. 1, 131 Iranian (65%) and 13 Afghan cases (22%) were susceptible to all 4 drug tested. The results showed that 72 patients (28%) were MDR-TB cases. Notably, 38 MDR-TB cases (52.7%) were isolated from afghan immigrants. Twenty patients (47%) had mono drug resistant strains (nine were INH, seven SM, three RF and one ETB monoresistant) and 22 (52%) had combined resistance. 62 patients (24%) had family history or were in contact with TB patients. HIV infection status was available for 98 patients (37%); out of which two were HIV positive (2.0%). Reviewing the patients questionnaires revealed that the crowded, poor-living condition (233; 93%), and low-salary (220; 85%) were common factors increasing the risk of developing TB (table. 1).

**Table 1:** Detail demographic data of the studied population.

	Non-cluster Patients (193 = 74.8%)		Cluster patients (65; 25%)	
Nationality	Iranian 151(78.2%)	Afghani 42(21.3%)	Iranian 48(73%)	Afghani 17(26%)

Mean age	47.91 ± 25	39.65 ± 11	44.17 ± 19	36.19 ± 8
Female	70(46.3%)	15(35.7%)	18(37.5%)	8(47%)
Male	81(53.6%)	27(64.2%)	30(62.5%)	9(56.9%)
Pulmonary	147 (97.3%)	37(88%)	47(97.9.%)	14(46.4%)
Extra-pulmonary	2(1.3%)	3(7.1%)	0	2 (30.7%)
Both	2 (1.3%)	2(4.7%)	1(2.0%)	1(1.5%)
AIDS	2(1.3%)Positive	17 (40.4%) Negative	3(6.25) Negative	17(26.1%) NA
	76(97.5%) Negative	25(59.5%)NA	41(85.4%) NA	
	73(48.3%) NA			
PPD	72(47%) Positive	16(38%) Positive	19(39.5%) Positive	4(23%) Positive
	18 (11.9%) Negative	5 (11.9%) Negative	8 (16.6%) Negative	3 (7.6%) Negative
	25(16.5%)equivocal	9(21.9%)equivocole	14 (29.1%)equivocole	7(11%) equivocole
	36(23.8%) NA*	12(28.5%) NA	7(14.5%) NA	5(29.4%) NA
History of family or close contact	27(17.8%)	17(40%)	10 (20.8%)	8(47.9%)
	96(63.5%)	5(11.9%)	35 (72.9%)	8(33%)
Susceptible	29(19.2%)	31 (73.8%)	5(10.4%)	7(41.1%)
MDR-Other resistant	26(17.2%)	6(14.2%)	8 (16.6%)	2(11.7%)
Crowded and low living condition	134(88.7%)	38 (90%)	44 (91.6%)	17(100%)
Low-Salary	129(85.4%)	40(95.2%)	35(72.9%)	16(94.1%)

*.NA, the data were not available

### IS6110-copy number

The copy number of IS*6110 *in each of the isolates was determined from the number of bands hybridizing the probes. Average number of IS*6110 *copies was 10.82 among Iranian and 11.8 among Afghan cases. The differences was not statistically significant (P > 0.05). Twenty one isolates (8.1%) contain 1–4 copies of IS*6110 *(Low-copy number), 218 isolates (88.3%) contain 6–15 copies and 31 isolates (12%) had more than 16 copies (high-copy number). Ten isolates (3.8%) had no copies of IS6110.

### Diversity of RFLP

In this study, 193 strains (74.8%) were infected with genetically different *M. tuberculosis *strains based on IS*6110*-RFLP pattern. 65 patients (25%) were clustered in 29 clusters. The size of clusters were ranging from 2 to 3 isolates, 22 clusters had 2 isolates (75.8%) and 6 clusters had 3 isolates (20.3%).

### Spoligopatterns

Thirty-six distinct spoligopatterns were observed. In total, 27 orphan patterns (10.4%) were seen and the remaining 231(89%) were contained within 9 superfamilies; EAI3(58; 25%), CASI(41; 17.7%), EAI4(32; 13.8%), T1(31; 13.4%), T2(16; 6.9%), HaarlemI (20; 8.6%), X1(12; 5%), Beijing(12; 5%) and CASII(9; 3.8%). Although, the diversity of these super families were different in cluster and non-cluster cases. As shown in table 2, the major superfamilies among non-cluster cases were EAI3 (30%), and CASI (18.6%), whereas in cluster cases were Haarlem I (27.6%), and Beijing (17%). The overall diversity of observed cluster was 0.13 (diversity = the number of shared types divided by total number of found isolates i.e., 36/258 = 0.13).

**Table 2:** The spoligopatterns in cluster and non cluster cases

**Spoligopatterns**	**Non-cluster cases (n = 193)**		**Cluster cases (n = 65)**		
	Iranian (n = 151)	Afghanis (n = 42)	Iranian (n = 48)	Afghanis (n = 17)	Total

EAI3	39(30.4%)	12(3.5%)	4(8.3%)	3(17.6%)	58(25.4%)
CASI	19(14.5%)	12(3.5%)	10(20.8%)	0(0%)	41(17.7%)
EAI4	26(20.3%)	3(7.8%)	2(4.1%)	1(5.8%)	32(13.8%)
T1	26(20.3%)	2(5.2%)	3(6.2%)	0(0%)	31(13.4%)
T2	8(6.25%)	0(0%)	7(14.5%)	1(5.8%)	16(6.9%)
Haarlem I	2(1.5%)	0(0%)	12(25%)	6(35.2%)	20(8.6%)
Beijing	1(0.78%)	0(0%)	7(14.5%)	4(23%)	12(5.1%)
X1	0(0%)	9(23.6%)	1(2.0%)	2(11.7%)	12(5.1%)
CASII	7(5.4%)	0(0%)	2(4.1%)	0(0%)	9(3.8%)

Total	128	38	48	17	231

Orphan cases	23(85.1%)	4(14.8%)	0(0%)	0(0%)	27

### Epidemiological studies of patients in clusters

**Figure 1:**
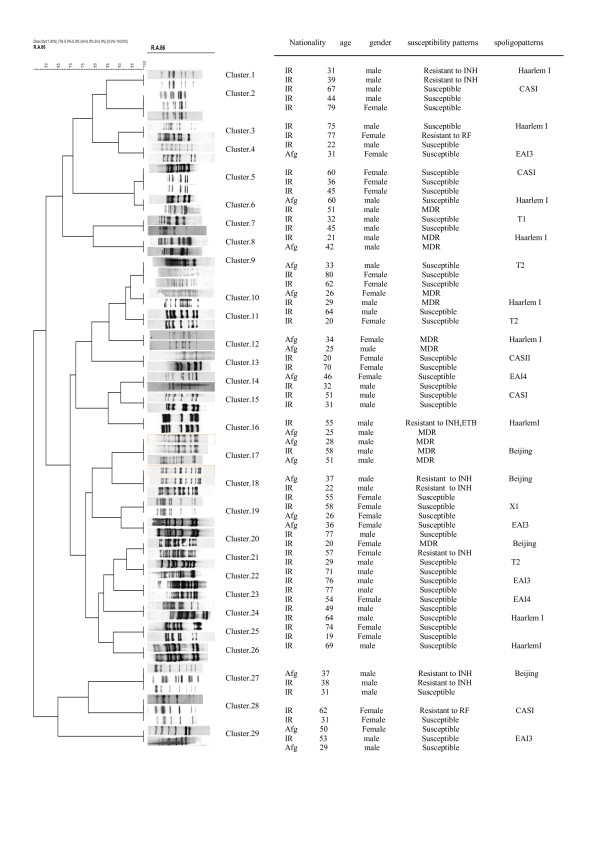
The figure show the intra-community transmission among Iranian and Afghan relapse cases.

65 *M. tuberculosis *isolates were clustered in 29 clusters. Sixteen clusters (55%) contained 34 Iranian TB cases and one cluster (3.4%) had two Afghan patients. The remaining clusters (12; 41%), were contained both native and Afghan TB cases (Figure. 1). Afghan patients in the clusters were immigrated in the last 10–15 years and all of them had one incomplete course of treatment in Afghanistan and one episode of TB in Iran. The mean of age in Iranian cases was 44.17 ± 19 (SD), whereas in Afghan cases was 36.19 ± 8 (SD) (P < 0.05). Reviewing the patient's questionnaires revealed that only 18 individuals (27.6%) had a family history or was in contact with TB patients (P > 0.05). Statistical analysis showed that patients in clusters were more likely to be male than female (1.6:1). The susceptibility results showed that 12 patients were MDR-TB cases, in which 7(58.3%) belong to afghan immigrants. A retrospective analysis found no direct transmission link between patients in clusters. Although, these patients developed their second episode of TB in the same period. It was also important to notice that all MDR-TB cases in clusters were belonged to Haarlem I and Beijing type of strains.

## Discussion

Using molecular typing, twenty-five percent of relapse cases (65; 25%) were clustered in 29 clusters. A retrospective studies revealed that patients in clusters developed their second episode of TB within the same period. Further analysis of spoligopatterns identified Haarlem I and Beijing types of *M. tuberculosis *strains in thirty eight percent of patients in clusters(38%)(table. 2). Therefore, the possibility of exogenous reinfection through transmission of particular *M. tuberculosis *strains were highlighted. Although, due to non-availability of previous culture genotyping results, we could not confirm the exogenous reinfection in them. Recently, investigators have suggested that the relative contribution of exogenous reinfection increases in parallel with the incidence of disease [6,8,18]. Most reported cases of exogenous reinfection observed among alcoholic residents of a homeless shelter or patients with advanced HIV infection [6,9,19]. We found no particular risk factors between patients in cluster and non-cluster cases. Retrospective analysis of cluster cases identified 41% of intra- community transmission between Iranian and Afghan TB patients. In our previous study, the impact of intra- community transmission was much lower (13.7%) than present result. Furthermore, we found that the Haarlem I and Beijing type of *M. tuberculosis *strains were the most frequent super families in intra-community transmission (figure. 1). The Haarlem I and Beijing strains have been reported in different geographical regions of the world and they thought to possess selective advantages in comparison to other *M. tuberculosis *super families [22,23]. Previously, we demonstrated that 44% of MDR-TB patients in intra-community transmission were belonged to HaarlemI (73%) and Beijing (27%) superfamilies [21]. Therefore, based on previous and present reports, it is clear that both Haarlem I and Beijing strains can cause epidemic and from epidemiological point it is necessary to conduct more extensive surveillance of MDR-TB strains because they might cause serious outbreaks [12]. During the year 2000–2005, 32% of initial TB patients that referred to our unit were from Afghan born immigrants [20]. Majority of these patients (58%) had either resistant to any drug or drug combination including MDR-TB [20]. In present report also, the rate of resistance to any drug or drug combination in Afghan patients were more than two folds as compared to Iranian (table 1). These finding highlighted the need to reinforce the TB policy measures with regards to screening immigrants from neighboring countries, which is absent in the current system. Based on IS6110-RFLP, 74.8% of patients were grouped in non- cluster cases and it was assumed that reactivation of endogenous infection remains the more probable cause of active tuberculosis, in studied populations (table. 1). Today, it is known that sterilization of a pulmonary lesion is possible through effective treatment regimens. But, it is also accepted that subsequent episodes of TB are almost invariably caused by endogenous reactivation of resistant strains [7,24]. In other words, majority of those who returned for treatment after default might develop resistant in comparison to those who returned after exogenous reinfection [7,9,25]. In this regards, we also found high number of MDR-TB strains among endogenous reactivation cases. The frequent superfamilies in MDR-TB cases of non-cluster cases were CASI (19; 31%) and EAI3 (12; 20%) (table. 2). Based on Sreevatsan *et al*, the EAI and CAS are belong to genetic group I organisms which are evolutionary older and they are the most frequent superfamilies in Central Asia and Middle East [26]. Our results also showed different susceptibility patterns for isolates in same clusters e.g., in the cluster numbered-27 (figure. 1); one strain was susceptible to all drugs tested and the other two strains were resistant to isoniazid. Mitchison [27] has already described the emergence of such drug resistance strains, solely due to irregularity in administration of drugs. That means the strains became acquired drug resistance isolates, as classically defined [24,25]. In the studied population, no relation was found between the patterns of IS6110-RFLP and susceptibility results. In fact, the number and manner of IS6110 positioning along the genome of *M. tuberculosis *does not have any relation with drug-susceptibility results [2,4]. Last but not the least, the major consideration of the usefulness of the IS6110-RFLP typing method is its specificity, which depends on the number of bands obtained. In the present study, 12% of collected strains had low copies of IS6110. Previously, we detected only 5.4% of strains with low IS6110 copy number [20]. Thereby, the prevalence of *M. tuberculosis *isolates with low or no IS6110 insert is not clear and further studies are required to show the real distribution of these strains within the country.

## Conclusion

Previous studies showed that more than 90% of active cases of tuberculosis in Iran resulted from reactivation of infection-contracted years before, and that recently transmitted diseases had a minor role. In this study, we also showed the higher rate of reactivation (74.8%) versus recent transmission (25.2%). Although, the incidence of intra- community transmission had a significant rises from 13% in 2005 to 41% in 2007. Therefore, it is necessary to adapt new strategies for rapid diagnosis, and efficient treatment of TB patients.

## Competing interests

The authors declare that they have no competing interests.

## Authors' contributions

PF design the work, carried out the RFLP analysis and draft the manuscript. MM did the critical review. MV carried out the patient's analysis. MM draft the manuscript. MA carried out the DNA extraction and susceptibility test and IS6110-RFLP. MK carried out the statistical analysis. PT carried out the patient's analysis. PB carried out the patient's analysis. MM carried out the patient's analysis. MB did the critical review. AZ did the critical review. AV design of study and did the critical review. All authors read and approved the final manuscript.

## Pre-publication history

The pre-publication history for this paper can be accessed here:


